# The Step Test Evaluation of Performance on Stairs (STEPS): Validation and reliability in a neurological disorder

**DOI:** 10.1371/journal.pone.0213698

**Published:** 2019-03-21

**Authors:** Anne D. Kloos, Deb A. Kegelmeyer, Katherine Ambrogi, David Kline, Meredith McCormack-Mager, Brittany Schroeder, Sandra K. Kostyk

**Affiliations:** 1 College of Medicine, Division of Physical Therapy, The Ohio State University, Columbus, Ohio, United States of America; 2 College of Medicine, Department of Neurology, The Ohio State University, Columbus, Ohio, United States of America; 3 Center for Biostatistics, The Ohio State University, Columbus, Ohio, United States of America; 4 College of Medicine, Department of Neuroscience, The Ohio State University, Columbus, Ohio, United States of America; University of Pennsylvania Perelman School of Medicine, UNITED STATES

## Abstract

**Background:**

Individuals with neurological disorders often have difficulty negotiating stairs that can lead to injurious falls. Clinicians lack a clinical tool to identify impairments in stair negotiation and to assist their decision making regarding treatment plans to improve stair performance and safety. We developed a new tool called the Step Test Evaluation of Performance on Stairs (STEPS) that is designed to assess stair performance and safety in neurological populations.

**Objectives:**

This study aimed to determine interrater and intrarater reliability of STEPS and its concurrent content validity to various clinical balance and mobility measures using individuals with Huntington’s disease (HD) as the first test population.

**Methods:**

Forty individuals with HD (mean age 50.35) participated. Three observers rated live performances of the STEPS (interrater reliability) and seven observers rated videotaped performances twice (intrarater reliability). STEPS scores correlated with clinical mobility and balance test scores.

**Results:**

Excellent inter- and intrarater reliability (ICCs = 0.91 and 0.89 respectively) and good internal consistency (α = 0.83) were found. Better STEPS performance correlated with better performance on co-administered motor and mobility measures and Stair Self-Efficacy scores. Per multivariable regression analysis, the Unified Huntington’s Disease Rating Scale modified motor score and descent time were significant predictors of STEPS performance.

**Conclusions:**

The STEPS tool is easy to administer, requires no special devices and can be completed in less than five minutes. In the HD test population, it shows high reliability and validity making it a potentially useful tool for assessing maneuverability and safety on stairs in HD. The results suggest that the STEPS tool warrants further study to determine STEPS cut-off values for fall prediction in HD and may prove useful as an assessment tool for other neurological disorders.

## Introduction

Stair ambulation is one of the more challenging motor activities of daily life. The ability to safely negotiate steps is an important component of the abilities needed to maintain mobility and independence both in the home and community. A recent study based on a large National Health Interview Survey identified stairs and steps as one of the three most common hazards associated with fall injuries across all age groups [1–2]. In our movement disorders clinic we have noted that patients with Huntington’s disease (HD) and Parkinson disease (PD) frequently report difficulties with stair negotiation or falls on stairs. While there are numerous clinical tests to assess gait and balance performance on flat surfaces (e.g., Tinetti [3], Timed Up and Go[4], 10-meter walk test [5]), there are no similar tests to assess performance on stairs. Specifically, there is no validated clinical assessment tool to provide clinicians with information to help identify gait impairments on stairs nor to guide clinicians’ decision making regarding treatment plans to improve stair performance and safety.

Stair navigation requires precise coordination of alternating limb movements for proper foot placement on each stair, high level stability control for single-limb balance during limb advancement, and volitional saccades for obtaining visual information about the characteristics of stairs [6–11]. To assess stair performance of our patients, we developed a tool called the Step Test Evaluation of Performance on Stairs (STEPS). The STEPS tool was constructed based on our clinical experiences and a review of stair negotiation outcome measures [3,12–16]. It has undergone several revisions to reach its present form based on our experiences using it and feedback from physical therapists (PTs). The test is easy to administer and takes only 2–5 minutes to complete.

We chose to validate the STEPS first in our HD population as this group is more homogeneous and as the diagnosis can be confirmed by genetic testing. A Danish study reported that 5.8% of deaths among 395 individuals with HD were due to accidents with 22% out of the 5.8% being from falls [17]. Individuals with HD have slowly progressive motor, cognitive and behavioral impairments that contribute to increased difficulty with stair negotiation leading to falls on stairs [18]. Specific problems related to stair navigation in HD include impairments in visuospatial processing, volitional saccades, balance, gait, cognition, impulse control, and insight into their deficits [19]. Various degrees of these deficits are similarly found in individuals with other neurological disorders. We hypothesized that assessment and treatment of stair negotiation deficits would need to consider these impairments with the expectation that a tool effective for HD would likely prove useful for individuals with other neurodegenerative gait disorders.

For the STEPS to be utilized to assess stair negotiation and fall risk, we needed to establish its reliability and validity in our HD test population. Therefore, the specific aims of this research were to determine the: 1) inter- and intrarater reliability and internal consistency of STEPS; and 2) concurrent content validity of the STEPS tool with commonly used balance and mobility tests in patients with neurological deficits. We also explored the association of physical and cognitive patient characteristics on STEPS performance to better understand underlying factors that impair stair performance in this population. We hypothesized that better STEPS scores would have moderate to good correlations with less severe physical and cognitive impairments.

## Methods

### Participants

The researchers recruited forty patients with a diagnosis of HD at their regular clinic visits during a six month period. Participants were ambulatory without needing physical assistance and cognitively able to consent to the study. Individuals with other neurologic or orthopedic diagnoses that altered walking were excluded. Participants signed informed consent and videotape release forms prior to participating in the study. The Ohio State University Institutional Review Board State University approved this study.

Demographic data including age, sex, years since diagnosis, CAG repeat size, and medications were obtained from the participants’ medical records. A neurologist (SK) certified in administering the motor section of the Unified Huntington’s Disease Rating Scale (UHDRS) [20] tested all participants on the motor examination and completed the Total Functional Capacity (TFC) assessment. The total possible score on the UHDRS motor scale is 124, with higher scores indicating worse performance. The TFC is a standardized scale used to assess capacity to work, handle finances, perform domestic chores and self-care tasks, and live independently. The TFC scale ranges from 13 (normal) to 0 (severe disability). The UHDRS cognitive battery sections (verbal fluency, symbol digit modalities test (SDMT), and Stroop word, color and interference tests) were administered by a trained nurse (KA). These cognitive tests measure executive functions, information processing, and visuospatial learning. Cognitive test scores are based on the number of correct answers and therefore higher scores indicate better cognitive performance. A PT (AK or DK) administered the timed 10-meter walk to measure gait speed, the Timed Up and Go (TUG) and the Tinetti Mobility Test (TMT) to measure mobility and balance, and the timed single leg stance test to measure static balance. Participants walked 10 meters (32.8 feet) for three trials and the time for the intermediate 6 meters (19.7 feet) was measured. They performed two trials of the TUG, which required them to stand up from a chair, walk 10 feet, turn around, walk back to the chair and sit down while they were timed. The TMT consists of balance and gait subscales, with higher scores out of 28 indicating better performance. To assess single leg stance, participants stood on one foot with their arms crossed at chest level or hands placed on hips for as long as possible without moving out of position or until they reached 30 seconds. Two trials were performed on each leg. Participants were asked if they experienced any falls on stairs in the past 6 months and in the past week. A faller was defined as a person who reported ≥1 falls during the previous 6 months. Participants completed the Stair Self-Efficacy (SSE) test which asked them to rate on a 0–10 scale (0- no confidence, 10 completely confident) how confident they were that they could go up and down stairs under different conditions and recover from a fall on stairs [14].

### STEPS

The STEPS tool consists of 16 items, with eight items each to measure performance in ascending and descending stairs (see S1 Appendix for instrument and instructions). Each item is rated using a scale ranging from 0–1 or 0–2, with a total possible score of 20 points. Higher scores indicate better performance.

### Interrater reliability

#### Raters

Raters were two PTs (DK, AK), and a clinic nurse (KA). The PTs with extensive experience using the STEPS trained the nurse on scoring the tool prior to starting the study. One of the PTs, designated the administering rater (AR), instructed the participants on what to do, guarded them and scored their performances. The other PT and nurse were observing raters (ORs) as their role was to watch with a frontal view at a 6 foot distance from the stair bottom and score each participant’s performance. Raters did not speak to each other during testing. This design was utilized to avoid excessive fatigue from performance of repetitive tests on one day. Testing participants on different days wasn’t feasible due to the time and expense for participants who travelled long distances or missed work to come to the clinic. This design was utilized previously in other validation studies using participants with amyotrophic lateral sclerosis [21] and PD [22].

#### Procedures

Participants performed one trial of the STEPS in a well-lighted stairway adjacent to the clinic with a flight of 10 standard height steps with bilateral handrails. The AR provided instructions to the participants (see Appendix 1) before they ascended and descended the stair flight. An OR used a stopwatch to measure the time for participants to ascend and descend the stairs. The ORs viewed participants’ performances without changing position. A camera was used to videotape performances. Testing took approximately 2–5 minutes. Participants could use any assistive and/or orthotic device they typically utilized for walking.

### Intrarater reliability

#### Raters

Two medical center staff PTs with experience treating individuals with neurological disorders and five first-year Doctor of Physical Therapy students were the raters. All raters received training on scoring the STEPS by the PT researchers (AK, DK) prior to starting the study.

#### Procedures

Each rater viewed and rated the videotaped test session for each of the participants and then repeated the same process one week later. Raters viewed each participant's taped performance only once without slowing or stopping the tape to simulate the observational conditions in the clinic and were not allowed to talk with each other Viewer ratings of videotaped performances of individuals have been used frequently in reliability studies [21–24].

### Statistical analysis

Interrater and intrarater reliability of the STEPS assessment scores were calculated using a random effects intra-class correlation coefficient (ICC) model of class 2. The inferred reliability from the ICC values were classified as follows: > 0.85 = excellent; 0.75–0.85 = good; < 0.75 = fair [25]. Cronbach’s alpha was calculated using the STEPS score breakdown from the AR with the alpha function in the psych R package [26]. Spearman rank correlation coefficients and 95% confidence intervals were calculated between the STEPS score (AR) and the UHDRS total motor examination score (UHDRS-TMS), timed 10-meter walk test, TUG, TMT, timed single leg stance test, and SSE test scores [27]. Spearman correlation coefficient evaluation criteria were: fair (0.25–0.50), moderate to good (0.50–0.75) and excellent (≥0.75) [27]. Spearman correlation was used as some associations appeared non-linear. Scatterplots were constructed with a Loess smoother to visualize the associations. The required level of significance for all tests was set at p < .05. Exploratory univariable linear regression was performed to examine the relationships between the STEPS score (AR) and specific physical and cognitive characteristics that the authors deemed most likely to affect stair performance. These characteristics included UHDRS modified motor (items 4–5, 8–15), eye movement (items 1–3), and chorea (item 7) subscale scores of the UHDRS motor section. The modified motor score (mMS) measures the ability to perform voluntary movements (e.g., finger taps, gait, tandem walking). The UHDRS eye movement score measures ocular pursuit, saccade initiation and saccade velocity; the chorea score measures chorea severity in the face, mouth, trunk, and extremities. Other variables were ascend/descend times, SSE test scores, and UHDRS cognitive battery test scores. A multivariable regression model was constructed based on variables found to be significant (p<0.05) predictors of the STEPS score in univariable regression. This model was then pared down based on its significant variables to achieve a final model.

## Results

Participant (25 female, 15 male) characteristics are shown in Table 1. According to TFC scores, 11 (27.5%) of the participants were in the early disease stage (TFC 11–13), 28 (70%) were in the middle stage (TFC 3–10), and one participant (2.5%) was in the late stage (TFC 0–2). Overall, STEPS scores averaged 15.5 with standard deviation of 4.1 and ranged from 4.0 to 20.0 with 25% of patients obtaining a score of 20. Six participants reported that they were not routinely exposed to stairs. Of the 34 patients who did report stair exposure, 11 (32%) were fallers. The fallers had STEPS scores that were on average 1.7 (difference = -1.7, 95% CI:[-4.42, 1.08], p-value = 0.24) points lower than the non-fallers. The average for the non-fallers was 16.2 (95% CI:[14.7, 17.8]) and for the fallers was 14.55 (95% CI:[11.4, 17.7]) Participants were on their usual medications which included anti-choreic, antipsychotic, antidepressant, and other medications.

**Table 1:** Participant characteristics.

	n	Mean ± SD (range)	Median
Age (yrs)	40	50.35 ± 13.63 (21–74)	51.50
Time since diagnosis (yrs)	34	5.45 ± 5.68 (0–21)	3.50
Total Functional Capacity	40	8.33 ± 3.07 (2–13)	8.00
UHDRS total motor score (0–124 possible)	40	34.03 ± 16.85 (0–66)	35.00
UHDRS modified motor score (0–52 possible)	40	13.58 ± 6.97 (0–52)	13.00
UHDRS eye movement score (0–24 possible)	40	7.33 ± 4.37 (0–14)	7.00
UHDRS chorea score (0 = 28 possible)	40	9.73 ± 5.37 (0–20)	10.00
UHDRS cognitive battery: Verbal Fluency Symbol Digits Modalities Test Stroop Color Stroop Word Stroop Interference	39	25.44 ± 11.52 28.39 ± 14.06 49.82 ± 17.06 58.00 ± 17.25 22.8 ± 9.31	24.00 27.50 50.00 54.00 22.00

Abbreviations: UHDRS, Unified Huntington’s disease rating scale.

### Reliability and internal consistency

One patient was missing a third rater and thus, only two were included in the interrater ICC calculation. Otherwise, all data were fully observed. The interrater intraclass correlation coefficient (ICC) for total STEPS scores between the three real time raters was 0.91 (95% confidence interval (CI) 0.86 to 0.95). The intrarater reliability score for the total STEPS scores recorded by the seven video review raters on Day 1 and one week later was 0.89 (95% CI 0.70 to 1). The Cronbach’s alpha value for the STEPS score was 0.83 (95% CI 0.75 to 0.91), indicating that the items of the STEPS score are measuring a similar underlying construct while not being redundant.

### Concurrent content validity

**Fig 1:**
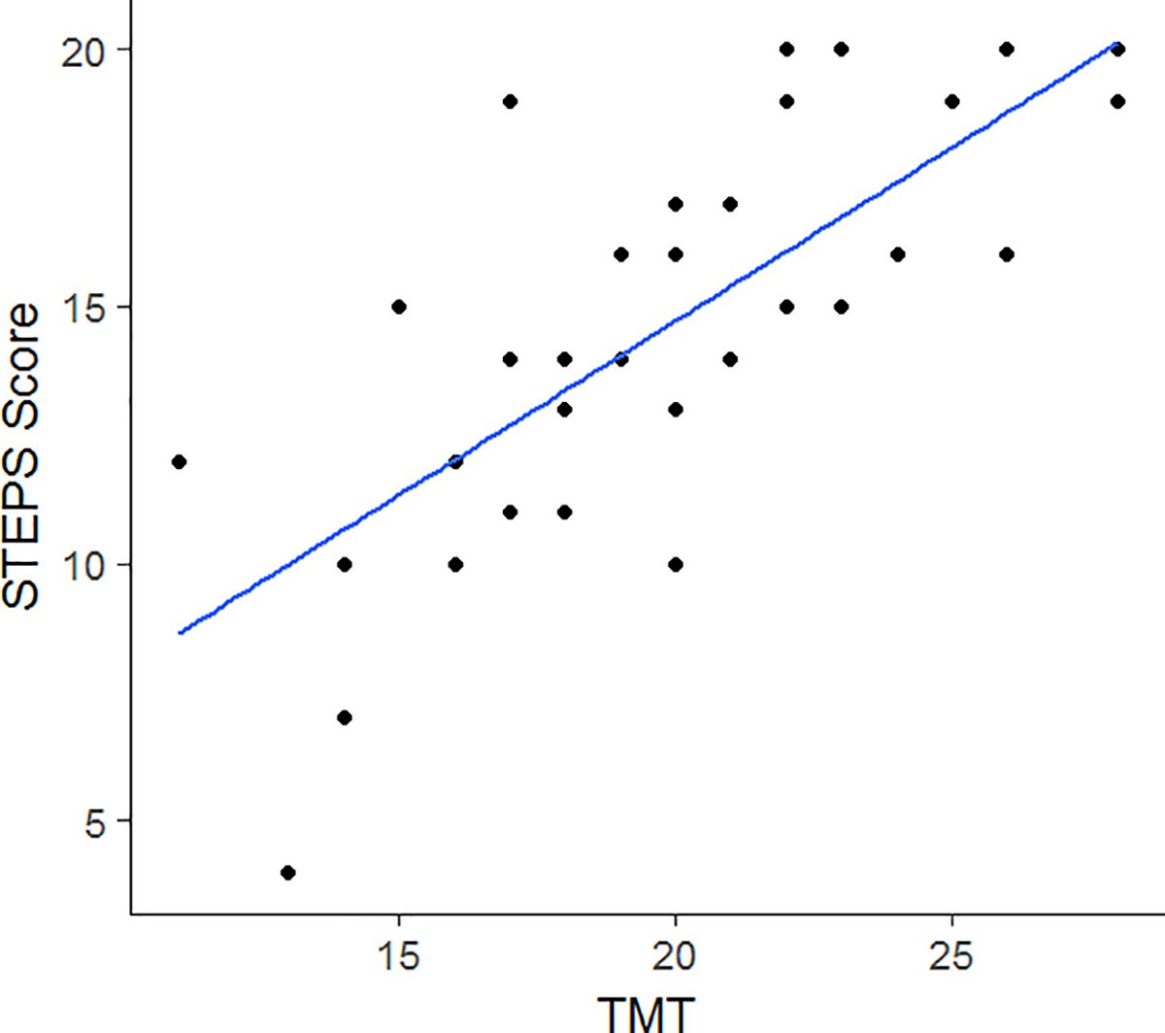
Univariable regression of Tinetti Mobility Test (TMT) versus STEPS scores. Higher scores on the TMT correlate with better gait and balance on flat terrain.

**Fig 2:**
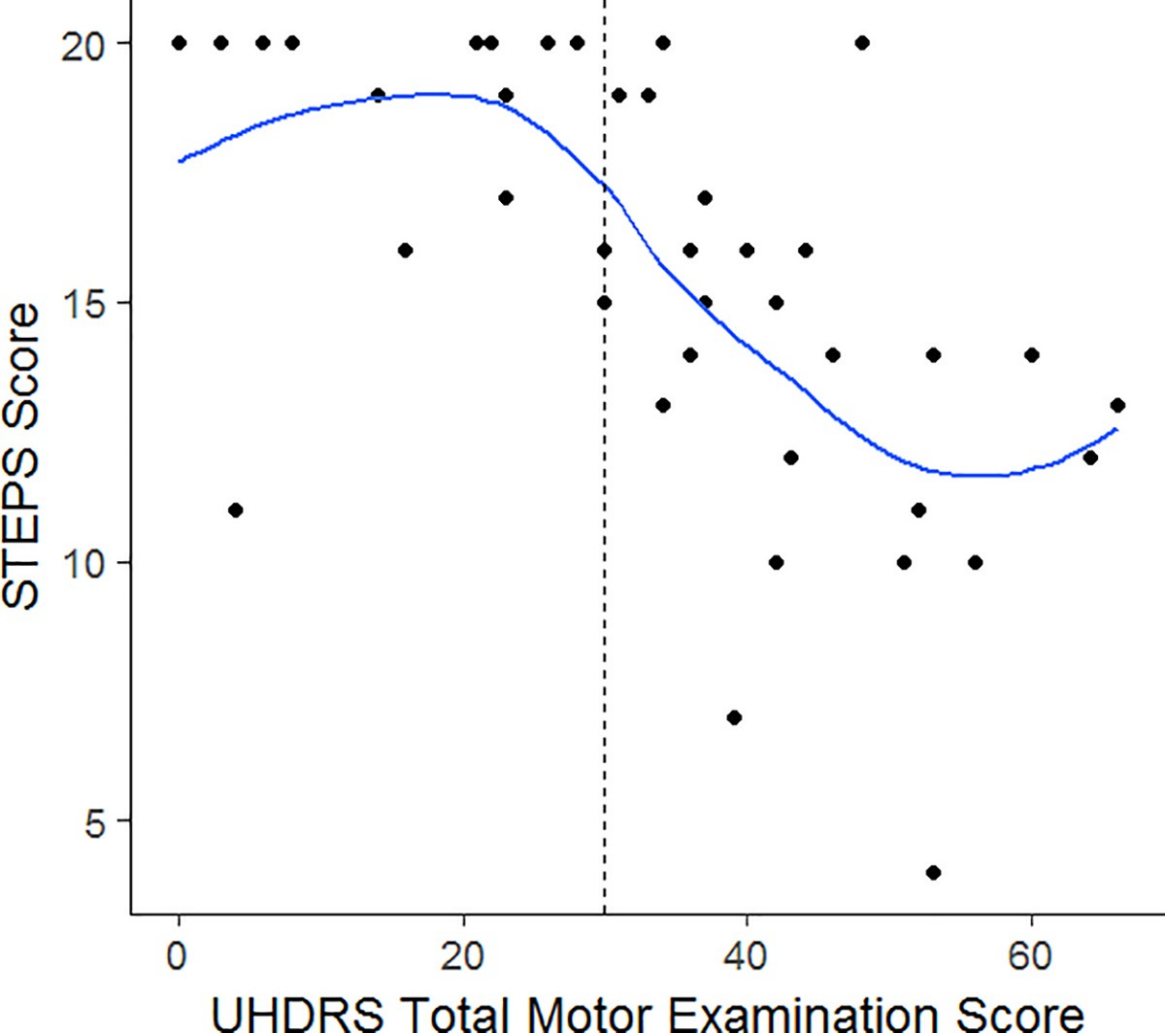
Scatterplot of total UHDRS motor examination scores versus STEPS scores with a Loess smoother. In general, performance on the STEPS was high until the UHDRS total motor score reached 30 above which declines in performance were seen.

Table 2 shows the correlation coefficients and 95% confidence intervals between the total STEPS score (AR) and various mobility and balance measures. All correlations were statistically significant with the highest correlation found between the STEPS and TMT scores (Fig 1). A scatterplot of the STEPS score and UHDRS-TMS revealed that STEPS scores remained high (16–20) until the UHDRS total motor score reached 30 when the STEPS performance scores began to decline (Fig 2).

**Table 2:** Correlations and corresponding 95% confidence intervals between total STEPS score (Administering Rater) and mobility and balance measures.

Factor	Correlation (Spearman)	Lower CI bound	Upper CI bound
UHDRS-TMS	-0.66	-0.81	-0.45
10-meter walk test	0.60	0.35	0.77
Timed Up and Go	-0.62	-0.78	-0.39
Tinetti Mobility Test	0.82	0.82	0.90
Single leg stance (right)	0.59	0.33	0.76
Single leg stance (left)	0.61	0.37	0.77
Stair Self-Efficacy test	0.60	0.35	0.77

Abbreviations: UHDRS-TMS, Unified Huntington’s Disease Rating Scale-total motor score.

### Association of physical and cognitive factors on steps performance

The results of the univariable linear regression analyses between STEPS scores and specific physical and cognitive patient characteristics are described in Table 3. One participant was missing verbal fluency, SDMT, and Stroop scores and was excluded. Variables found to be significant (p <0.05) predictors of the STEPS score were the UHDRS mMS, UHDRS eye, and chorea subscale scores, ascend and descend times, SSE test, and Stroop word and interference scores. Using these significant variables, the multivariable regression model analysis (R^2^ = 0.65) found that the UHDRS mMS (coefficient (95% CI), -0.12 (-0.21, -0.03), p = 0.0085) and the descend time (-0.38 (-0.50, -0.26), p <0.001) were the only significant predictors, and both had an inverse relationship to the STEPS score.

**Table 3:** Single-variate linear regressions on total STEPS scores (Administering Rater) and physical and cognitive variables.

Factor	Coefficient (95% CI)	p-value	R^2^
Age	-0.08 (-0.17, 0.14)	0.094	0.07
UHDRS mMS	-0.21 (-0.33, -0.09)	**<0.001**	0.26
Eye Score	-0.39 (-0.66, -0.11)	**0.007**	0.18
Chorea Score	-0.37 (-0.58, -0.15)	**0.002**	0.24
Ascend time	-0.41 (-.60, -0.23)	**<0.001**	0.34
Descend time	-0.44 (-0.56, -0.31)	**<0.001**	0.58
Stair Self-Efficacy test	0.11 (0.06, 0.15)	**<0.001**	0.38
Verbal Fluency	0.095 (-0.11, 0.30)	0.35	0.02
Symbol Digit Modalities Test	0.04 (-0.13, 0.21)	0.61	0.01
Stroop Word	0.19 (0.04, 0.33)	**0.015**	0.15
Stroop Color	0.17 (-0.04, 0.35)	0.069	0.09
Stroop Interference	0.14 (0.003, 0.28)	**0.045**	0.10

Abbreviations: UHDRS mMS, Unified Huntington’s Disease Rating Scale modified motor scale. Bolded values are significant.

## Discussion

STEPS is the first clinical tool developed specifically to assess stair performance in individuals with neurological disorders. This study examines the reliability and validity of the STEPS tool in HD, a slowly progressive neurodegenerative disorder. Our findings show that the STEPS has excellent inter- and intrarater reliability (ICCS = 0.91 and 0.89 respectively) and good internal consistency (α = 0.83). The correlations between STEPS scores and validated clinical balance and mobility measures are moderate to excellent, demonstrating that the test has good concurrent content validity. Lower UHDRS mMSs and times to descend stairs predicted better STEPS performance scores. Taken together our findings suggest that the STEPS is a valid tool to assess stair performance in ambulatory individuals with HD.

Our findings demonstrate that the STEPS can be administered with high levels of interrater and intrarater reliability, and that the items are generally measuring stair performance. Interrater reliability was higher than intrarater reliability which may be related to difficulty seeing all aspects of performance from only one vantage point viewing videotaped performances. In the analysis of internal consistency, the foot clearance item contributed the least to score variation due to the high number of “normal clearance” scores on ascent and descent, suggesting that it may not be a necessary component of the STEPS score in the HD population. However, foot clearance is likely to be more relevant for assessing stair safety when the STEPS tool is used in other neurological disorders. Our clinical experience monitoring patients with PD on stairs was a key reason for including foot clearance when designing this tool.

Better performance on the STEPS was moderately to strongly correlated with better performance on common clinical measures of mobility and balance. The highest correlations were between the STEPs scores and TMT scores, suggesting that both tests measure constructs of postural control and mobility. The observation that STEPS scores declined in individuals with UHDRS motor scores ≥ 30 suggests that the sum of increased involuntary (chorea, dystonia) and voluntary (Luria, finger taps, gait) movement impairments are negatively impacting stair performance in this population. This conjecture is supported by previous findings that UHDRS motor scores > 42 are associated with significant disability and fall risk [28]. An advantage of using the STEPS tool is that it provides clinicians with information about the impact of motor impairments on stair performance beyond what is captured by UHDRS-TMS scores alone. This information may help define individualized therapy interventions. An additional advantage of the STEPS tool is that it can be administered by all clinicians who might see patients with HD including those who are not trained or certified to perform the UHDRS motor exam. The STEPS tool can easily be administered by physical therapists and other care providers as a screening tool for stair performance and safety for HD and may also be a useful tool for other neurological disorders. The moderate correlations between STEPS scores and comfortable gait speed (10-meter walk), TUG, and SLS scores were not surprising, since the majority of items on the STEPS tool were specifically designed to measure different aspects of gait and balance function than are included in these tests. Participants’ perceptions of their stair performances moderately reflected their performance on stairs per the SSE scale. Hamel and Cavanaugh [14] stated that elderly individuals who had lower SSE scores were more likely to ascend and descend stairs at a slower speed, use the handrails, and position themselves closer to the rail. We have noted that some individuals with HD continue to descend stairs rapidly despite exhibiting loss of balance and/or reporting previous falls on stairs; this is perhaps due to a lack of insight into their deficits as anosognosia is common in HD [29]. Clinicians may want to determine if there is a mismatch between stair confidence (SSE) and performance (STEPS) measures, and if found, implement interventions to bring them into better alignment.

Individual physical and cognitive variables that predicted STEPS performance were the UHDRS mMS, eye, and chorea subscale scores, ascend/descend times, SSE test, and Stroop word and interference scores. Bradykinesia and impaired coordination as measured on the UHDRS mMS may cause hesitation, inaccurate foot placement, and discontinuous ascent and descent of stairs. Young and older adults spend the majority of time during stair walking looking at the stairs, and gaze fixations on stair edges may provide important cues for proper foot placement and balance [30]. Delayed initiation and/or slowness or absence of eye saccadic movements in individuals with HD may decrease their ability to obtain important visual cues from the environment. Chorea can contribute to balance and walking difficulties that may negatively affect stair performance [31]. One might postulate that dyskinesia in patients with PD might similarly affect stair performance and STEPS scores.

Faster ascend/descend times in general predicted better STEPS performance in HD. We did not integrate ascend and descend times in the STEPS tool itself because we did not want the tool to be dependent upon the number of steps assessed since the numbers of steps available at clinical sites for using this tool may vary. However, the significant association of STEP scores with ascend/descend times suggests that timed measures may be worth doing simultaneously with STEPS, especially for assessing decreased function and increased fall risks over time and to assess intervention effects. In a study of community dwelling elderly changes in ascent and descent time over three steps predicted overall functional decline even in those with normal overground gait speeds [32].

The Stroop word reading and interference subtests were the only cognitive tests measured in this study that predicted STEPS performance in individuals with HD. The word reading test has been identified as one of the strongest markers of HD progression [33]. The interference test is a measure of executive functions, particularly selective attention and cognitive flexibility and processing speed [34]. We previously observed that Stroop word and interference tests correlate with TMT scores (r_p_ = 0.45, p <0.001) in a gait analysis study of 70 patients with HD [35]. Clinicians evaluating stair performance in individuals with HD and other neurodegenerative disorders should consider the possible contribution of cognitive impairments to stair performance deficits.

The UHDRS mMS and descend time were the only significant predictors of STEPS performance in HD from the multivariable regression analysis. For every 1 point increase in the mMS there was a 0.12 decrease in the STEPS scores and every 1 second increase in descent time meant a 0.38 decrease in STEPS scores. Voluntary movement deficits in individuals with HD steadily progress throughout the disease and lead to functional limitations [36]. Individuals with HD at our clinic frequently report that they have more difficulty going down stairs than up, and that they fall more often going down stairs. Studies in older adult populations have shown that people exhibit more cautious behavior, more handrail use, and fall more frequently during stair descent than ascent [9,14]. A primary cause of falls in the elderly during stair descent is inadequate leg extensor eccentric muscular force to control the lowering of the body’s center of mass [9]. It is unknown whether a similar mechanism may be occurring in people with HD.

A limitation of this validation study is that it was used at only one site and on a relatively small number of ambulatory individuals with HD in the early to middle stages of the disease. We were not able to reliably determine a fall risk cut-off value for the STEPS due to the low numbers of participants who reported falls on stairs during the prior 6 months in this volunteer study population. There may have been a reluctance of those who felt most uncomfortable on stairs to volunteer to participate. It is also possible that the lack of difference in STEPS scores between fallers and non-fallers was caused by differences in the amount of stair use. The individuals most likely to fall and have worse STEPS scores may have compensated for their lack of stair confidence or abilities by decreasing or avoiding stair use to lessen their opportunities to fall. The utility of this tool to describe stair performance in clients who are high functioning with low disease burden may be limited due to the potential for a ceiling effect. We noted that 25% of those who participated in this study obtained the highest possible score of 20. There may be a ceiling effect in which performance on stairs of clients who are high functioning with low disease burden cannot be described by this tool.

In conclusion, our findings indicate that the STEPS tool is a reliable and valid tool for assessing stair performance in ambulatory individuals with HD. It is easy to administer, requires no additional equipment and takes only 2–5 minutes to complete. Future studies with larger numbers of individuals are needed to determine cut-off values for fall prediction in the HD population and to determine the reliability and validity of the STEPS in other neurologically impaired populations such as individuals with PD, multiple sclerosis, Alzheimer’s disease or stroke.

## Supporting information

S1 Appendix STEPS: Step Test Evaluation of Performance on Stairs. (DOCX)

S1 Dataset Raw data for Table 1. (XLSX)

S2 Dataset Raw data for Table 2. (XLSX)

S3 Dataset Raw data for Table 3. (XLSX)

S4 Dataset Raw data for Fig 1. (XLSX)

S5 Dataset Raw data for Fig 2. (XLSX)
