# Supplementary material for: The Step Test Evaluation of Performance on Stairs (STEPS): Validation and reliability in a neurological disorder
Source: PLoS One. 2019 Mar 21;14(3):e0213698. doi: 10.1371/journal.pone.0213698 (PMC6428278; doi:10.1371/journal.pone.0213698)
Supplement: S1 Appendix — (DOCX) [file pone.0213698.s001.docx]

**S1 Appendix. STEPS: Step Test Evaluation of Performance on Stairs**

© Ohio State University 2018

| Ascend | score |
| --- | --- |
| 1. Hesitation upon initiation or transition of first two steps  0 = hesitation  1 = no hesitation |  |
| 2.Stepping pattern (self-selected)  0 = Step-to pattern or sideways pattern chosen/unable to reciprocate upon further instruction  1 = Step-to pattern or sideways pattern chosen/able to reciprocate upon further instruction  2 = Reciprocal/step-through pattern |  |
| *Score the following items while having the individual ascend the stairs using reciprocal pattern if he/she is able. If unable, score based on pattern used.* | |
| 3. Foot clearance  0 = not clearing or missing a step; toe drag  1 = normal clearance |  |
| 4. Foot placement  0 = irregularity with variability of foot position and base of support; feet are externally rotated to widen base of support  1 = precise and controlled foot placement; stays in midline, symmetric |  |
| 5. Ascent Balance  0= bilateral assist (handrail or person; any combination) and/or assistive device; constant need of 1 hand rail or assist of person  1 = occasional need of assist (handrail or person)  2 = no use of assist (handrail or person) |  |
| 6. Continuity of ascension  0 = discontinuous steps/extended pause on one leg  1 = continuous steps; equal time spent on each leg |  |
| 7. Ability to self-propel up stairs  0 = requires assist to propel self up to next step (e.g. pushing with upper extremity, pulling on handrail, assist from another person or multiple attempts)  1 = able to fully power up independently, without using upper extremity or pulling on handrail; no assist from other person. |  |
| 8. Trunk stability  0 = unstable (exaggerated trunk lean in frontal and/or sagittal planes  1 = stable (minimal trunk lean any plane) |  |
| Descend | score |
| 9. Hesitation upon initiation or transition of first two steps  0 = hesitation  1 = no hesitation |  |
| 10.Stepping pattern (self-selected)  0 = Step-to pattern or sideways pattern chosen/unable to reciprocate upon further instruction  1 = Step-to pattern or sideways pattern chosen/able to reciprocate upon further instruction  2 = Reciprocal/step-through pattern |  |
| *Score the following items while having the individual descend the stairs using reciprocal pattern if he/she is able. If unable, score based on pattern used.* | |
| 11. Foot clearance  0 = not clearing or missing a step  1 = normal clearance |  |
| \| 12. Foot placement  0 = irregularity with variability of foot position and base of support; feet are externally rotated to widen base of support  1 = precise and controlled foot placement; stays in midline, symmetric \| \| --- \| |  |
| 13. Descent Balance  0 = bilateral assist (handrail and/or person; any combination) and/or assistive device; constant need of 1 hand rail or assist of person  1 = occasional need of assist (handrail or person)  2 = no use of assist (handrail or person) |  |
| 14. Continuity of descent  0 = discontinuous steps/extended pause  1 = continuous descent; equal time spent on each leg. |  |
| 15. Ability to control Descent  0 = knee buckles or uncontrolled lowering is observed  1 = controlled lowering on every step |  |
| 16. Trunk Stability  0 = unstable (exaggerated trunk lean in frontal and/or sagittal planes)  1 = stable (minimal trunk lean in any plane) |  |
| TOTAL ASCEND (10) |  |
| TOTAL DESCEND (10) |  |
| **TOTAL (20)** |  |

General Instructions:

The test should be administered on stairs with standard height steps [approximately 7 inch (17.78 cm) rise and 11 inch (27.94 cm) run] and with at least one handrail. The number of steps is not critical to this tool, but we recommend at minimum being able to observe individuals negotiate three or more steps.

Have the client stand close to the first step and give the client the following instructions:

“I want you to climb the stairs as you normally would. Try not to use the handrail, but if you need to use it you may. When I say “GO,” climb to the top of the stairs and then stop.”

At the top of the stairs have the client turn and face the steps. Then give the instructions “I want you to go down the stairs as you normally would. Try not to use the handrail, but if you need to use it you may. When I say “GO,” go down the stairs and then stop.”

**Ratings should be made based on the worst performance observed. For example, if you observe the behavior one time, then the lower score is given.**

Instructions for individual items:

**Items 1 and 9 Hesitation**: Observe the client at the moment you tell the client to “go” for both ascending and descending the stairs. If there is a hesitation or any pause ≥ 1 second prior to starting to climb the stairs, then this is scored a “0.” If the client stops to talk to you or otherwise becomes distracted from the task, restart by giving the instructions again and, say “go” again.

**Items 2 and 10 Foot pattern**: Instruct the client to ascend/descend as he/she normally would. If the client is climbing with a step-to pattern (i.e., step up with one foot and then step up with the opposite foot to the same step), ask the client to try a step over step pattern if he/she is able. If the client is able to do step over step (i.e., step up with one foot and then step up with the opposite foot to the step above the other foot) at least once on each leg without physical assistance, then score this item a “1,” If the client uses a step-to pattern on one leg score this item a “0.”

**Items 3 and 11 Foot Clearance**: Any instance of foot dragging or failure to clear a step on the first try is scored a “0.”

**Items 4 and 12 Foot Placement**: These items assess how accurate foot placement is on the step above or below. Note that a client whose progress up or down the stairs is not in a straight line would be placing the feet out of midline or irregularly and should be scored a “0” on this item. Placing the feet outside of hip width or with feet externally rotated to widen the base of support is scored a “0.” Note: Do not take off points if the person has a fixed foot deformity that causes the person to have his/her feet in an externally rotated position. If placement is inconsistent, varying from step to step, score this a “0.”

**Items 5 and 13 Balance**: Occasional touching the handrail or the person guarding during ascent or descent results in a score of 1; use of the handrail and touching the person guarding would be equivalent to bilateral handrail use and scored a “0.” Use of assistance for greater than ¾ of the ascent/descent is equivalent to constant use and results in a score of “0.”

**Items 6 and 14 Continuity**: If the timing of the client’s ascent and descent is at a steady, even pace, score “1.” Any slowing or speeding up indicates discontinuity and would be scored as a “0.”

**Items 7 and 15 Control**: These items require observation of the client’s ability to propel the body up or down stairs using knee extension alone. If the client requires multiple attempts to step up the score is “0.” Use of the arm to assist in lifting the body up or to control the descent of the body is also scored a “0.” If the person guarding has to assist to help lift the body to ascend a step or to control descent or the knee appears to “buckle” it is also scored a “0.”

**Items 8 and 16 Trunk Stability**: Observe the trunk for excessive forward, backward, or side to side leaning or sway. The presence of any excessive trunk movement or leaning is scored a “0.”
